# *In vivo* STED microscopy visualizes PSD95 sub-structures and morphological changes over several hours in the mouse visual cortex

**DOI:** 10.1038/s41598-017-18640-z

**Published:** 2018-01-09

**Authors:** Waja Wegner, Alexander C. Mott, Seth G. N. Grant, Heinz Steffens, Katrin I. Willig

**Affiliations:** 1 Optical Nanoscopy in Neuroscience, Center for Nanoscale Microscopy and Molecular Physiology of the Brain, University Medical Center Göttingen, Göttingen, Germany; 2 Collaborative Research Center 889, University of Göttingen, Göttingen, Germany; 3 Max Planck Institute of Experimental Medicine, Göttingen, Germany; 4 Genes to Cognition Program, Centre for Clinical Brain Sciences, Chancellor’s Building, University of Edinburgh, Edinburgh, EH16 4SB UK

## Abstract

The post-synaptic density (PSD) is an electron dense region consisting of ~1000 proteins, found at the postsynaptic membrane of excitatory synapses, which varies in size depending upon synaptic strength. PSD95 is an abundant scaffolding protein in the PSD and assembles a family of supercomplexes comprised of neurotransmitter receptors, ion channels, as well as signalling and structural proteins. We use superresolution STED (STimulated Emission Depletion) nanoscopy to determine the size and shape of PSD95 in the anaesthetised mouse visual cortex. Adult knock-in mice expressing eGFP fused to the endogenous PSD95 protein were imaged at time points from 1 min to 6 h. Superresolved large assemblies of PSD95 show different sub-structures; most large assemblies were ring-like, some horse-shoe or figure-8 shaped, and shapes were continuous or made up of nanoclusters. The sub-structure appeared stable during the shorter (minute) time points, but after 1 h, more than 50% of the large assemblies showed a change in sub-structure. Overall, these data showed a sub-morphology of large PSD95 assemblies which undergo changes within the 6 hours of observation in the anaesthetised mouse.

## Introduction

The postsynaptic membrane of excitatory glutamatergic synapses contains an electron dense thickening known as the post-synaptic density (PSD). The individual proteins in the PSD are assembled into a hierarchy of complexes and supercomplexes^1–3^. PSD95, a member of the membrane-associated guanylate kinase (MAGUK) family, is a scaffold protein that assembles ~ 1.5 MDa supercomplexes of glutamate receptors, ion channel complexes and signalling proteins^1,3–5^. PSD95 supercomplexes are essential for controlling synaptic strength and synaptic plasticity in response to patterns of neural activity^6,7^, which lead to changes in spine and PSD size^8–10^ and raise the possibility that PSD95 organisation is dynamic.

Electron microscopy (EM) has shown that PSDs appear as distinctive, approximately circular discs of 180 to 750 nm (mean 313 nm) in diameter^11^. Another EM study estimated the PSD size distribution of layer 2/3 pyramidal neurons from mouse visual cortex at an age of 8–12 weeks to be very broad, ranging from 0.01 to 0.33 µm^2^ (average 0.08 µm^2^)^9^. So far EM is the preferred technique to measure the PSD size because of its unprecedented resolution; however, EM cannot image dynamic morphometric changes of the PSD. Live-cell light microscopy of the expression of a fluorescent protein fused to PSD95, which is one of the most abundant scaffolding proteins in the PSD, has been used to examine the PSD. By using this technique, it was shown *in vivo*, that spine stability is related to the appearance of PSD95 on the heads of dendritic spines^12^. Although newly formed spines that acquired a PSD are not necessarily converted into persistent spines, spines that persisted over a number of days typically had stable PSDs^12,13^. Although persistent spines and their PSDs are relatively stable from the second postnatal week onwards, PSD95 molecules have a rapid turnover rate^13^. In young mice of postnatal day 10–21 (P10-21) the retention time was in the range of 60 min, and this increased with developmental age to about 100 min (P70). The time PSD95 molecules remain in the PSD is therefore much shorter than the half-life of the PSD95 molecule itself, which is in the range of 8 h–5 d depending on the technique or the source of PSD95 (synaptic fraction *in vitro* or total brain *in vivo*, Alvarez-Castelao *et al*.^14^ and ref. within). With confocal microscopy it was shown that PSDs undergo rapid (within 10 minutes), continuous morphometric changes in cultured neurons^15^.

All of these light microscopy studies bear two limitations: First, overexpressing PSD95 results in mature synapses^8,16^ which might not reflect their natural state, and second, the diffraction limited resolution of conventional light microscopy which cannot resolve morphological details of the PSD. With the advent of a whole family of superresolution light microscopy techniques such as STED, RESOLFT, PALM, STORM, etc.^17^, protein sub-structures in the living environment have become accessible. As such, PALM has been used to resolve the organization of rescued PSD95 in live neurons showing that these proteins are enriched in domains of ~ 80 nm, and are dynamic on a time-scale of minutes (not specified in detail)^18^. Broadhead *et al*. utilized g-STED and PALM microscopy of PSD95-eGFP or PSD95-mEos2 fluorescent knock-in mice^19^ to perform a large-scale analysis of sub-clusters of PSD95 which showed diverse characteristics in different sub-regions of the hippocampus. While this approach has overcome both limitations of earlier approaches by using an endogenous label and novel light microscopy techniques, the tissue analysed was fixed and morphological changes were not detectable.

Here we present the *in vivo* STED microscopy of PSD95 morphology in layer 1 of the visual cortex of living anaesthetised adult mice over an imaging period of 6 h. So far, STED microscopy is the only superresolution light microscopy technique applied to a living mouse^20–22^ as it is ideal for tissue imaging and possesses high imaging speed. We found that the morphology of PSD95 is highly diverse; large assemblies showed a complex sub-structure of ring-like or horse-shoe shapes, clustered or smooth morphology or arbitrarily assembled nanoclusters. These sub-structures were much more diverse than previously described and morphed over time; after 1 h more than 50% of the large assemblies underwent a morphological change and 90% within our imaging period of 6 h.

## Results

### *In vivo* STED microscopy of PSD95 assemblies

**Figure 1:**
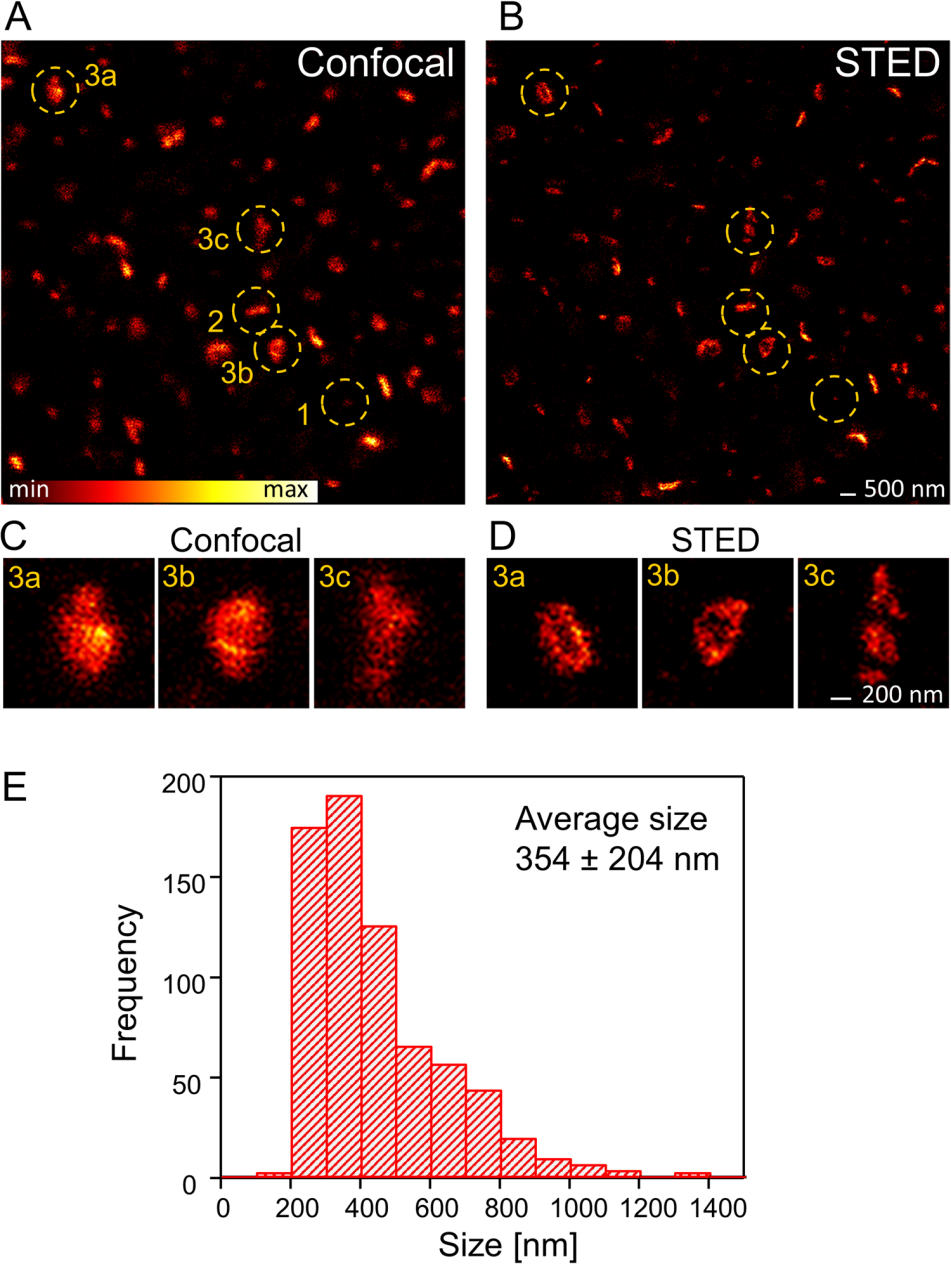
PSD95 shape and size determination *in vivo*. (**A**) Confocal and (**B**) STED microscopy image of PSD95-eGFP in L1 of the binocular mouse visual cortex (6 µm below the cover glass). The PSD95 morphology is very diverse: Some spots are very small and dark (1), some are elongated, stripe-like (2), and some are large protein assemblies (3a–c). (**C**) Magnification of large protein assemblies marked in (**A**), imaged with confocal microscopy. (**D**) Superresolution STED microscopy of the same assemblies as in (**C**) reveals a complex sub-structure which is not visible in the confocal image. 3a and 3b show a ring-like morphology whereas 3c shows three distinct clusters. (**E**) Absolute frequency histogram of PSD95 assembly size analysed from *in vivo* STED microscopy images, average size ± SD. 708 assemblies of n = 4 mice were analysed.

We used homozygous knock-in mice expressing endogenous PSD95 fused to eGFP under the control of all native regulatory elements^19^. The expression level of PSD95-eGFP in these mice is comparable to that of wild type PSD95 and the mice did not show changes in electrophysiological properties^19^. Adult mice (P80–115) were anaesthetised and a craniotomy was performed. A 3 mm round opening was drilled into the bone above the visual cortex and closed with a cover glass. The side of the cover glass facing the brain was sparsely coated with fluorescent beads to measure the depth of the imaging plane in reference to the cover glass. During the whole experiment the mouse was kept on a heating plate and vital functions were recorded (see Methods for detail). After the surgical procedure the mouse was transferred to a home-built upright STED microscope, adapted to superresolve eGFP labelled protein structures. In the whole field of view the fluorescence labelling was very dense and PSD95 assemblies could only be distinguished with a confocal microscope (Fig. 1A). Confocal microscopy visualizes PSD95 assemblies of various brightness; some spots were very dark and just detectable (Fig. 1A“1”), other spots were elongated (Fig. 1A“2”) and a few were large PSD95 assemblies (Fig. 1A“3”). STED superresolution microscopy (Fig. 1B) revealed the assemblies in better detail; small spots (such as Fig. 1A“1”) were noticeably smaller in the STED image as in the confocal image which indicates that the size of this spot is below the diffraction limited resolution of the confocal microscope. Large assemblies (Fig. 1A“3a–c”) revealed a sub-structure in the STED microscopy image (Fig. 1B) which was not visible in the corresponding confocal image. A magnification of these assemblies showed that some assemblies appear ring-like (Fig. 1D, “3a,b”) or showed distinct clusters (Fig. 1D,“3c”) which is morphological information not achievable from the confocal image (Fig. 1C). In the following study we set out to characterize the morphology and plasticity of these protein assemblies in previously unknown detail within the living anaesthetised mouse.

### Size of PSD95 assembly

Spines are attached to the dendrite in multiple orientations with a broad variation of shapes. The PSD is not necessarily attached to the tip of the spine but can be at various orientations^9^. With an average PSD diameter of 313 nm^11^ and length of 17 nm of vertical filaments associated with PSD95^23^, we expected a disc-like shape of the PSD95 assemblies. Therefore, PSDs which were aligned along the optical axis, i.e. z-axis, appeared as stripes or elongated spots in the image, whereas PSDs which were within the focal plane, i.e. the xy-plane, showed the surface view. The point-spread function of our *in vivo* STED microscope provides only superresolution within the focal plane and is diffraction limited along the optical axis (~500 nm); as such, assemblies of distinct PSD95 sub-structures not in the focal plane cannot be reliably analysed using our 2D STED microscope. To determine the size distribution of PSD95 assemblies *in vivo*, however, it was necessary to include all of the measured spots into the analysis. As such, we categorized and analysed the PSD95 assemblies imaged with STED microscopy in the following way: For small rounded spots (Fig. 1B “1”) which were around the diffraction limit or smaller; we could not detect the orientation and therefore measured only the diameter. Elongated spots (Fig. 1B“2”) indicating a more or less perpendicular alignment of the PSD95 disc were measured in length. These length measurements are an estimate of the diameter as we cannot resolve the 3D shape; but averaged over many assemblies, it gives an estimate of the average diameter of the randomly oriented PSD95 disc. Large protein assemblies were mostly perforated (Fig. 1D,“3a and b”) or clustered (Fig. 1D,“3c”) and not usually round (details of the morphology are discussed in the next paragraph). To indicate a diameter for these complex shapes we encircled these assemblies and fit it with an ellipse; for a size estimate, we averaged the small and large axis of the ellipse. For more details cf. methods. All sizes were pooled and displayed in a frequency histogram which shows a broad size distribution ranging from 0.1 µm to 1.5 µm with an average of 354 nm (Fig. 1E). The distribution is asymmetric with a long tail and therefore the median of 290 nm is lower than the average size. These values are in good agreement with EM measurements of PSDs of 313 nm in diameter^11^ and an area of 0.08 µm^2^ (320 nm diameter) of layer 2/3 neurons^9^. Interestingly, the size of the PSD95 assemblies does not sufficiently correlate with the brightness of the assembly which is sometimes used as a size estimate (Supplementary Fig. S1). Although we used a superresolution technique, the values are still the size of a convolution of the PSD95 assembly with the microscopes point-spread function (PSF). The size of the effective PSF of the STED microscope is very sensitive to aberrations due to a refractive index mismatch of the brain tissue. We used the correction collar of the glycerol immersion objective to compensate for spherical aberrations^20^. For quantification of the resolving capability of our microscope we estimated the PSF of the *in vivo* STED microscope directly in the recorded images. Line profiles (average of 3 lines) were measured at small structures in the STED microscopy image and fit with a Lorentzian function (Supplementary Fig. S2). An average of 84 nm full-width at half-maximum (FWHM) was determined. In an earlier study we determined a 43–70 nm resolution with a similar microscope and STED laser power but with the yellow fluorescent protein^24^. 84 nm is an upper estimate due to the finite size of the PSD95 protein assembly and the attached eGFP. However, a resolving power of <84 nm is clearly smaller than the average PSD95 assembly (354 nm) and the size measurement (Fig. 1E) provides a good approximation of the size of PSD95 assemblies in layer 1 of the visual cortex.

### Morphological changes of individual large PSD95 assemblies

While the small assemblies of PSD95 did not show any kind of sub-structure, and the elongated spots of PSD95 are likely perpendicular to the imaging plane which cannot be dissected with our 2D STED microscope, we concentrated on the large protein assemblies which predominantly showed a complex sub-structure of various shapes. We set out to image the morphological changes of these large PSD95 assemblies and to quantify the percentage undergoing a morphological change. We recorded z-stacks of 30 or 40 µm x/y STED microscopy images with 3 to 5 z-slices at different positions of the cranial window. With the coordinates of the motorized x/y translation stage we were able to re-acquire all positions even after a number of hours. For fine adjustment of the imaging area and to check for drift, we recorded a confocal overview image marginally out of the imaging area. In general the mouse preparation and microscopy setup was very stable and the repeated images had an offset of a few micrometres which could be easily corrected by moving the mouse according to the confocal reference image before the STED image and for fine tuning by image alignment. At each coordinate, we also recorded a confocal x/z image to determine the depth of the image plane below the cover glass which was treated with fluorescent beads. Each coordinate was assigned a time interval of 0.5 h, 1 h, 2 h, 3 h etc. after which we moved the mouse to the respective coordinate and recorded a STED microscopy image stack. This was repeated 3–4 times before photobleaching limited the signal to noise ratio. Using this methodology we were able to study morphological changes at different time intervals, without the need to record too many images at the same position and therefore minimized any potential damage. To maximize the signal to noise ratio and number of time points, we reduced the STED power, resulting in a slight reduction in the resolution of this experiment compared to Fig. 1. To record a base line and to study the potential influence of the light on the PSD95 assemblies, we also repeated STED microscopy imaging at short intervals of ~1 min.

**Figure 2:**
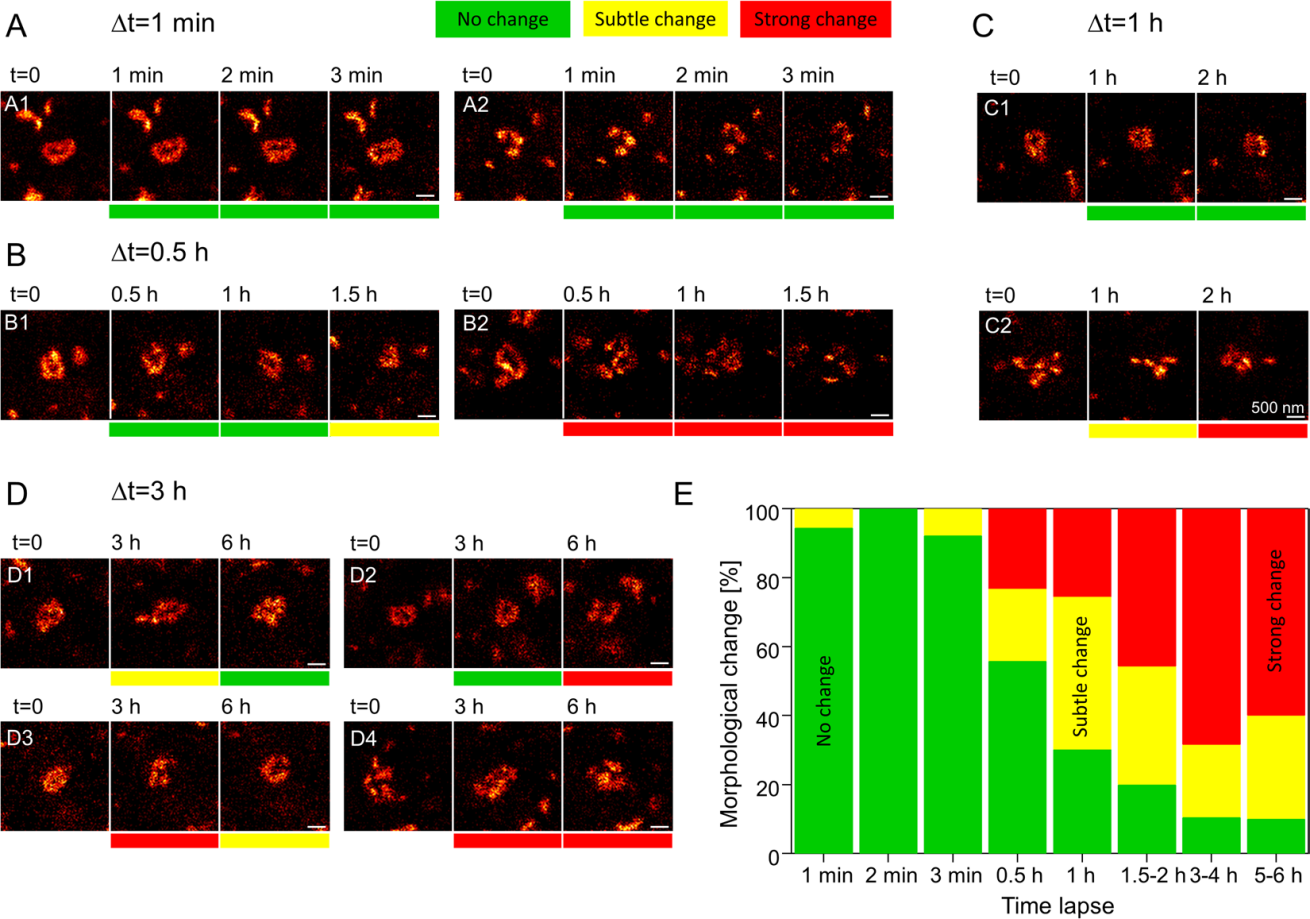
Morphology of large PSD95 assemblies changes within hours. (**A**–**D**) STED microscopy image of a selection of large protein assemblies recorded at different time intervals in the visual cortex of an anaesthetised mouse. The morphology is very diverse; many assemblies are smooth ring-like (A1, B1, B2 at t = 0, C1, D1, D2, D3), some are clustered (A2, B2 at 1.5 h, C2, D4); the cluster can be arranged in horse-shoe (A2), ring-like (C2 at 2 h, D4 at 6 h) or arbitrary (B2 at 1.5 h, C2 at 0 h, D4 at 0 h) shape. Shape changes were categorized as no changes (green), subtle changes (yellow) or strong morphological changes (red). All changes refer to t = 0. (**A**) Within minutes the morphology is not changing. (**B**) After 0.5 h assemblies can be rather stable (B1) or change strongly (B2). (**C**) Over 2 h assemblies can be stable (C1) or undergo morphological change (C2). (**D**) Assemblies can change and reverse to the original structure (D1), are stable after 3 h and changed after 6 h (D2), or undergo multiple changes (D3, D4). (**E**) Stacked histogram of relative frequencies of morphological changes of PSD95 assemblies; changes refer always to t = 0. Number of protein assemblies analysed: 18 (1 min), 18 (2 min), 13 (3 min), 43 (0.5 h), 43 (1 h), 35 (1.5–2 h), 19 (3–4 h), 10 (5–6 h) of n = 4 mice. All analysed protein assemblies can be found in the supplementary data. Scale bars 500 nm.

For analysis, we inspected all STED image stacks and selected those large PSD95 assemblies which met the following criteria: We chose only large PSD95 assemblies appearing mainly in a single plane, i.e. which are parallel to the focal plane, to ensure that we could access the sub-structure with our 2D STED microscope; therefore we omitted assemblies in the first and last image frame of the z-stack. We compared the surrounding PSD95 expression pattern to ensure that the same assembly was analysed at the different time points. For short time intervals of up to 1–2 h, most of the assemblies could be re-acquired. After long time intervals of 5–6 h the PSD95 pattern was changing; the image area could still be identified by landmarks such as outstanding assemblies or blood vessels but only a few PSD95 assemblies could be clearly re-acquired. Some PSD95 assemblies had moved relative to the surroundings and could not be relocated. (All analysed 104 assemblies are included as Supplementary data). The morphology of the assemblies that were relocated showed various different shapes (Fig. 2A–D, representing only a selection of analysed assemblies). Many assemblies were ring-like or doughnut-shaped with a PSD95 depleted central region (A1, B1, B2 at t = 0, C1, D1, D2, D3). Sometimes the morphology of the assemblies was horse-shoe like (A2, D3 at 6 h), with a few assemblies resembling an 8-shape or double-ring (B2 at 0.5 h). The sub-structure is often smooth and continuous (A1) but can also be composed of clusters (A2, C2, D4). The cluster can be arbitrarily distributed (C2) or sorted in ring-like (C1) or horse-shoe (A2) structures. Whether the assembly is continuous or clustered can also alter over time, e.g. D3 is rather continuous at 0 h and clustered after 3 h, D4 is clustered at 0 h and rather smooth after 3 h. The variability of shapes is very high and does not afford the possibility of sorting the assemblies into distinct sub-classes, as a limited number of sub-classes could not account for the high variability. Instead, we analysed the strength of their morphological changes over time which could be associated with variations in synaptic strength. Assemblies which did not change their overall morphology but show minor modifications, such as a smooth ring-like structure which appears more clustered, but remains ring-like after a time interval (D1 at 3 h) or a small movement of a sub-cluster (C2 at 1 h), were classified as a “subtle change” and marked it yellow (Fig. 2). Changes of the overall morphology such as a ring-like shape changing to an 8-like shape (B2 at 0.5 h) were characterized as “strong change”, marking it red. Assemblies without a distinct morphological change were attributed “no change” and marked green (A1). To be able to pool our data of different time intervals and different number of time points at which an assembly could be relocated, we classified the change of shape of all assemblies with regard to their shape at t = 0 and merged all relative changes in a stacked histogram (Fig. 2E). For a short time interval of 1 min no major changes were observed; the assemblies A1 and A2 (Fig. 2A) did not change over 3 min and in total only a single “subtle change” at 1 min and 3 min was observed (Fig. 2E). Within 0.5 h intervals stable morphologies were observed that showed subtle changes after 1.5 h (Fig. 2B1) or strong changes after 0.5 h, as well as after 1 and 1.5 h (B2). In summary, out of all investigated assemblies 56% were stable, 21% had subtle changes and 23% had strong changes after the first 0.5 h. At the 1 h interval we also observed no changes (C1) or subtle and strong changes (C2). Pooling all data recorded after 1 h shows 30% with no change, 44% with a subtle change and 26% having a strong change. At 3 h intervals an assembly was stable for 3 h and showed a strong change after 6 h (D2) or strong changes after the first 3 h (D3, D4). Interestingly, some assemblies were morphing back into the original structures (D1) or similar structures as at the beginning (D3) after 6 h. Pooling the data for changes after 1.5–2 h revealed 20% do not change, 34% showed a subtle change, and 46% a strong change. After 3–4 h 11% did not change, 21% showed a subtle change and 68% a strong change. Similarly, after 5–6 h 10% did not change, 30% showed subtle changes and 60% displayed strong changes. Taken together, this *in vivo* STED time lapse analyses reveals that the strength of the morphological change is highly variable, an assembly can fall apart into clusters (B2), reverse the morphological change (D1) or might remain stable over 6 h (Fig. 2E, 10% no change).

### Ensemble mapping of PSD95 assemblies over hours

**Figure 3:**
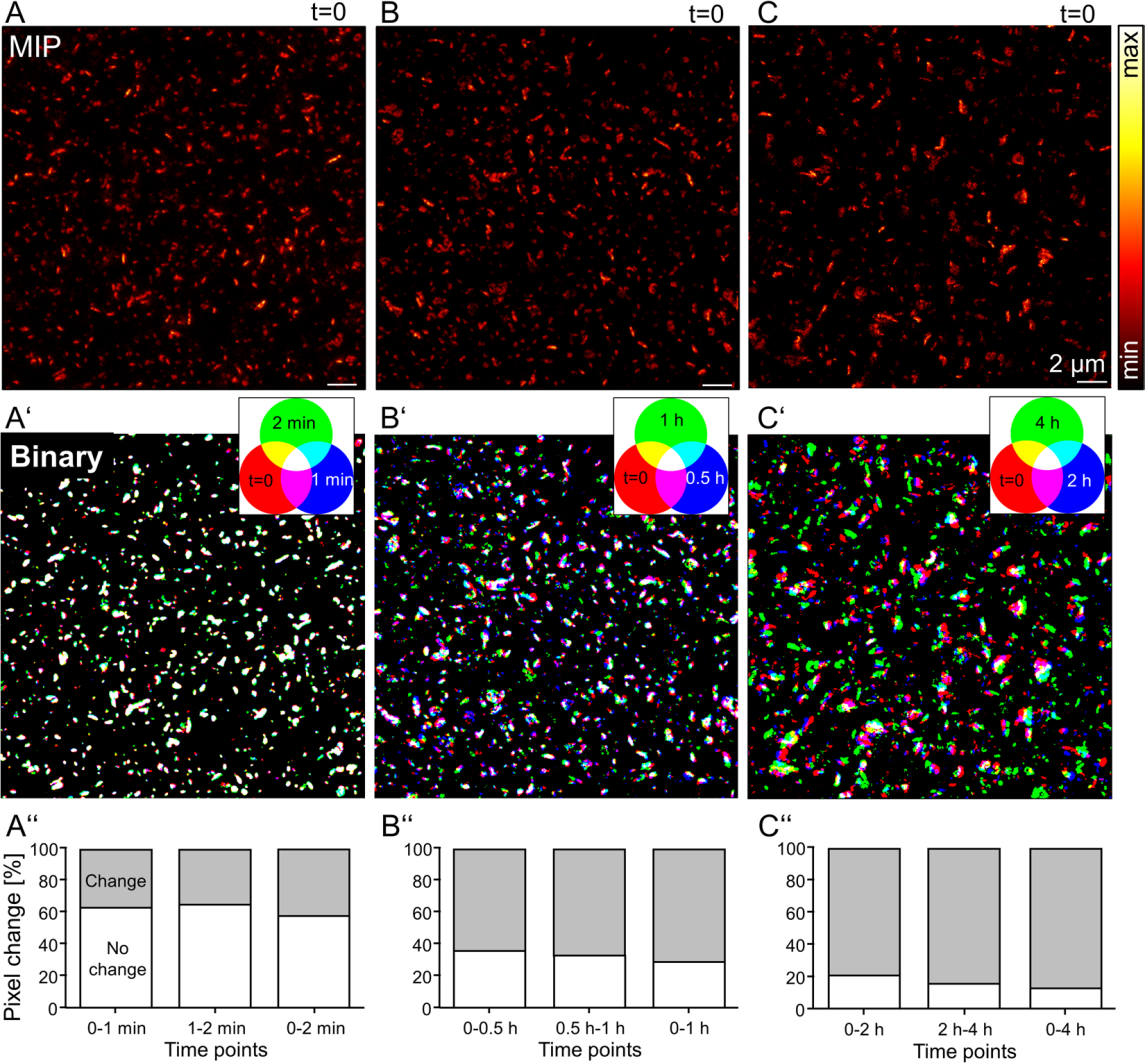
Mapping PSD95 assemblies over several hours in an anaesthetised mouse. (**A**,**B**,**C**) Maximum intensity projections of STED microscopy z-stacks at different areas in the visual cortex which were repetitively imaged at different time intervals of 1 min, 0.5 h and 2 h. (A’) RGB overlay of three binary images created from maximum intensity projections (MIP) at time point 0 (shown in A) (red), after 1 min (blue) and after 2 min (green). (B’) RGB overlay of binary images of MIPs recorded at t = 0 (created from B) (red), t = 0.5 h (blue), and t = 1 h (green). (C’) RGB overlay of binary images of MIPs recorded at t = 0 (created from C) (red), t = 2 h (blue) and t = 4 h (green). Overlapping colour areas are as indicated on each colour wheel. (A”–C”) Percent of pixels which do not change between two time points averaged over the whole binary image above. (A”) ~60% of the pixel of all assemblies in A’ at t = 0 overlap with the assemblies at t = 1 min (0–1 min) and correspondingly between t = 1 min and t = 2 min (1–2 min) and t = 0 min with t = 2 min (0–2 min). (B”) Only <40% of pixels of the assemblies in B’ show an overlap between 0 and 1 h, and ~20% after 4 h (C”). Scale bars 2 µm.

The large PSD95 assemblies analysed above are a small number (n = 104) of the overall assemblies. Most of the assemblies were small and did not show a sub-structure or were not parallel to the focal plane (Fig. 1). If we cannot analyse the structure of most assemblies, is there a way to assess the stability of the whole PSD95 ensemble? We chose image stacks which were recorded at different time points and which overlapped well in the z position. First, a maximum intensity projection (MIP) was created for STED microscopy image stacks of 3 different positions in layer 1 of the visual cortex (t = 0, Fig. 3A,B,C) as well as for the time points after 1 and 2 min (of area Figs. 3A), 0.5 and 1 h (of area Fig. 3B) and 2 and 4 h (of area Fig. 3C), respectively. The MIPs were smoothed and binary images were generated resembling an outline of the assemblies. The binary images of the different time points were aligned in x and y. Assigning the three time points as red, green, and blue (RGB) colour and merging the three colour channels by colour mixing showed how the assemblies were moving relative to each other or changed their morphology (Fig. 3A’,B’,C’). The white areas represent overlapping pixels at all 3 time points; red, green, and blue areas denote the presence of pixels at only one time point; and cyan, yellow, and magenta denote pixels overlapping at two time points. After 1 min time intervals very little change could be observed and the majority of all 3 time points overlap; indicated by most assemblies shown as white (Fig. 3A’). For the 30 min time intervals (Fig. 3B’) the RGB overlay revealed more translational movement of the assemblies; general changes to yellow, magenta, and cyan from white indicate that the assemblies were moving between each time point in x and y or some assemblies might disappear by moving out along the z-axis. The large assemblies are often shown in white colour indicating limited movement. For small clusters of 100–200 nm, however, it was much less likely that an overlap was observed at the differing time points, than that of large assemblies with a size >1 µm. For the larger time intervals of 2 h the overlay of binary images (Fig. 3C’) showed clearly that the same area was imaged, making it possible to re-align the images. These images showed a similar pattern where several assemblies were observed to be at the same position. However, a high number of assemblies were also observed to move or change their morphology as indicated by their red, blue or green colour. To quantify the changes between the different time points the number of pixels of all assemblies which overlapped between two time points was analysed. The overlapping area was then normalized with the whole area covered by all assemblies at both time points. From 0 to 1 min and 1 to 2 min 63% and 65% of the pixels overlapped, with 58% remaining stable for the entire 2 min period (Fig. 3A”). After a time interval of Δt = 0.5 h only 36% were stable after the first interval, 33% after the second interval and 29% after a total of 1 h (Fig. 3B”). Following Δt = 2 h only 21% did not change in the first interval and 16% in the second (Fig. 3C”). After 4 h 13% of the area was still overlapped (Fig. 3C”). In summary these images showed that the position of PSD95 assemblies were stable over a shorter minute time scale but started to move the position and shaped after 0.5 h. Some assemblies were very stable in position and shape indicated by the white colour (Fig. 3B’) while others moved or changed their shape (blue, red, green colour). After larger time intervals the movement and shape changes increased and fewer assemblies were stable (reduced white colour, Fig. 3C’). Unfortunately, due to the high density of assemblies within the visual cortex it was not possible to further track the movement of all assemblies. As such, additional labelling of the dendritic spines would help to identify the same assemblies over time in future.

## Discussion

Currently, the preferred technique for measuring the sub-structure of PSD morphology is through the use of EM, but this carries its own limitations due to the need for samples to be fixed before analysis. Here we have utilized *in vivo* STED microscopy to visualize not only increased detail of the PSD, through analysis of PSD95, but also observed the plasticity of PSD95 morphology within the living mouse visual cortex, allowing us to move from fixed samples to the living environment. By passing the sub-diffraction limit we have determined the size of the PSD95 distribution in layer 1 of the visual cortex in the anaesthetised mouse. With an average of 354 nm the size distribution shows a long tail up to 1.5 µm. The majority of large PSD95 assemblies oriented planar to the image plane showed a complex sub-structure. Often we observed a ring-like structure which was either a continuous distribution of PSD95 or arranged into nanoclusters. Some of the observed shapes where horse-shoe shaped, whilst others consisted of randomly arranged clusters. This complex morphology was not static but changing over time; after 1 h more than 50% of the assemblies showed an observable change. The morphing of the PSD95 assemblies did not occur continuously; some assemblies where stable over 3 h but then changed after another 3 h, others changed and then regained their original shape. Within our imaging time course of 6 h 90% of the large PSD95 assemblies underwent a morphological change. Alignment of MIPs of different time points showed the changes of the whole ensemble of assemblies; most assemblies changed their positions relative to each other after 2–4 h (Fig. 3C’, non-white) as well as altered their shapes.

In order to analyse PSD95 assemblies, homozygous PSD95-eGFP transgenic mice were used. We have analysed morphological changes of 2–8 assemblies per image stack in detail, which constitutes a minute fraction of the total assemblies. The size of the analysed large assemblies ranged from 500 to 1300 nm with an average of 796 nm (Supplementary Fig. S3) and therefore matches the tail of the PSD95 size distribution in Fig. 1E. It is therefore highly probable that similar complex sub-structures are also present throughout, but are currently inaccessible with our 2D STED microscope. However, large PSD95 assemblies which are not parallel to the image plane are expected to be equivalent to the selection we dissected in Fig. 2. STED microscopy can in principle be used for 3D improvement in resolution over the diffraction limit but this technique is more prone to tissue induced aberrations and therefore has not yet applied to the living mouse brain.

Electron microscopy studies often discriminate between macular, i.e. homogenous without interruption and perforated PSDs^9,25,26^. This is very similar to our data, although we visualized only PSD95, a sub-protein of the PSD. Of all 104 analysed large assemblies we observed only one macular PSD95 assembly which converted to a ring after 5 h (Supplementary data), whilst all others showed a disruption and therefore were regarded as perforated patches. Small spots (cf. Figure 1B) did not show a sub-structure and were therefore macular; however, we could not rule out the possibility that this was due to our resolution limit of ~ 80 nm. Previous super-resolution studies of PSD95 have always analysed a clustered distribution of PSD95^19,27^. These studies analysed all assemblies and did not differentiate between vertical and parallel alignment of the PSD disc and therefore might have overlooked the complex sub-structure, which we think is not rare but with a 2D superresolution microscope only visible when parallel to the image plane. Although most of the previous work was done in fixed samples, the description of PSD95 accumulating in a nanocluster distribution is not due to fixation induced clustering. MacGillavry *et al*.^18^ previously identified PSD95 sub-clusters by using live-cell superresolution PALM microscopy and moreover our *in vivo* STED data clearly shows that there are sub-types of PSD95 nanoclusters within the intact visual cortex.

Furthermore, we have shown that the PSD95 assemblies change their morphology over time. We categorized these changes as none, subtle, or strong. It is obvious that this classification is very subjective and therefore 4 of the authors evaluated the data independently first. We noted that although each individual was applying a different threshold for the different classes the overall result was the same: The morphology of large PSD95 assemblies is stable during the minute time points, but starts to change after 0.5 h, and increases with investigation time; at our maximum time interval of 5–6 h 60% of the investigated assemblies showed a strong change. At this time we can only speculate on the impact of these changes on the synaptic machinery and therefore did not quantify it further. A more quantitative analysis of our observation would require an interpretation which is not possible without further investigation. However, could the morphological change be induced by the imaging light? Working with volume or actin labelling previously has shown us that damage such as blebbing always appears quicker than our fastest imaging interval of 1 min. We did not observe any marked changes in the 1 min time intervals and therefore we do not expect light induced change to occur. Another indication that what is observed is a baseline rather than an actively stimulated change is that the assemblies were morphing back into the original structure (Fig. 2D1). Moreover, the changes were not unidirectional, which would indicate stimulation; assemblies fall apart into clusters (Fig. 2B2) or, opposite, clusters form a continuous assembly (Fig. 2D4). However, we cannot completely rule out light induced changes and will in the future implement further controls, e.g. visual stimulation to induce the morphological change. The previously cited work, done by Blanpied and colleagues (confocal microscopy), revealed changes in PSD95 morphology occurring in fewer than 10 minutes^15^. It should be noted that this work is not directly comparable to our study as it was performed using dissociated neurons prepared and cultured from E18 rat hippocampus (investigated at 3–6 weeks)^15^. Within our current study we have focussed on the *in vivo* live environment of layer 1 in the primary visual cortex in P80–115 anaesthetised mice, where a mature intact complex neuronal network is present. While a useful tool, neuronal cell culture has several limitations when compared to the live animal. The neurons used by Blanpied *et al*. were seeded at a density of 6 × 10^4^ cells/cm^2^ ^15,28^ which is much lower than the densely packed neuronal network in the living mouse visual cortex (>9 × 10^6^ cells/cm^2^)^29^. Ivenshitz and Segal showed that neurons in spares cultures expressed larger bursting activity with longer durations compared to dense cultures^30^. The levels of phosphorylation and palmitoylation may also vary between cultured cells and the living mouse, as it is a dynamic process regulated by diverse inputs. This is also important, as PSD95 requires e.g. threonine-19 phosphorylation for mobilization, through reduced membrane association, which is correlated with NMDA stimulation^31^ or serine 561 phosphorylation which has been shown recently to increases PSD95 dynamics at the synapse^32^. N-terminal palmitoylation of PSD95 is essential for the interaction with the membrane and ion channels^33,34^. It is therefore possible that these variations could lead to differences observed between cultured neurons and our *in vivo* study.

But what could be the functional implication of this morphology? PSD95 is known to be one of the most abundant scaffolding proteins in the PSD^35^, organizing a family of postsynaptic signalling complexes, including adhesion proteins (neuroligin), ion channels, AMPA, and NMDA receptors^1,4,5,10^. PSD95 has been shown to stabilizes surface expression of NMDA receptors by binding to its GluN2B subunit as well as binding to the negative regulator STEP61, promoting its degradation^36^. Via an interaction with stargazin, PSD95 has also been shown to bind to AMPA receptors, and as such a AMPA receptor complex is anchored at the postsynaptic membrane by palmitoylation of PSD95^34,37,38^. The dynamic distribution of PSD95 has therefore a direct implication on trapping AMPA receptors to the synapse and by palmitate-mediated recycling PSD95 regulates the synaptic strength^10,38^. By using immunolabelling with EM tomography, Chen *et al*. were able to show that AMPA and NMDA receptors are not homogenously distributed throughout the PSD as it was previously assumed, and additionally both receptors are found in separate nanodomains with different stoichiometry regarding its vertical binding partner PSD95 (NMDA-PSD95 1:2, AMPA receptor-PSD95 1:1)^23,39^. NMDA receptors-clusters were found to be more prominent in the central region of the PSD, whereas AMPA receptors tended to be more localized at the periphery^23^. Knockdown of PSD95 induced the loss of AMPA receptors in the PSD as well as other scaffolding proteins, but does not influence NMDA receptors^23^. We assume that the morphology and the changes observed are part of the neuroanatomical diversity which is a fundamental part of synaptic activity, although the clearly defined mechanism is still to be investigated.

For a more detailed understanding of these morphological changes it would be interesting to correlate the PSD95 morphology with the spine shape, and try to induce these changes through stimulation. Previous studies have shown that PSD95 size correlates with the size of the spine^12,40^. Therefore we assume that the large assemblies belong to mushroom spines, which indeed needs to be proven in the future by an additional labelling of the spine morphology in the living mouse. This could be verified by using either a cytosolic spine volume-, or actin labelling^21^. However, we can assume that the spine head is at least in the size of the PSD95 assembly. Labelling of the spine would also facilitate the visualization of the fate of PSD95 assemblies for longer time intervals (>6 h), making it easier to distinguish between PSDs appearing or disappearing during the imaging period. An additional benefit would be the implementation of 3D into our analyses, as well as going forward to chronic imaging revealing longer time intervals.

## Methods

### Mouse generation and genotyping

PSD95-eGFP mice were bred to homozygosity and genotyped as described^19^.

### Mouse surgical procedure

All mouse experiments were performed according to the guidelines of the national law (Tierschutzgesetz der Bundesrepublik Deutschland, TierSchG) regarding animal protection procedures and approved by the responsible authorities, the Niedersächsisches Landesamt für Verbraucherschutz und Lebensmittelsicherheit (LAVES, Oldenburg, Germany).

General anaesthesia was initiated by i.p. injection of 60–80 mg pentobarbital sodium (in 0.9% NaCl) per kg body weight. Once anaesthetised, the left jugular vein was cannulated and the anaesthesia was continued, with 75 mg·kg^−1^·h^−1^ methohexital sodium (Brevimytal®, HIKMA, Gräfelfing, Germany) i.v. throughout the duration of the experiment. A tracheotomy was performed to intubate the mouse with a T-shaped tube for artificial ventilation. The skin around the tracheotomy was then closed with suture clips and the mouse positioned in a prone position. The mouse was paralyzed with pancuronium bromide (6 mg·kg^−1^·h^−1^, Actavis, Langenfeld, Germany) and connected to artificial ventilation to avoid movements by active respiration. Depending on the weight, the mice were ventilated at 100 to 120 strokes per minute and breath volume of 100 to 140 µl. Expiration was passive and controlled by a solenoid valve at the respiration gas output. A mixture of 50 vol % N_2_, 47.5 vol % O_2_ and 2.5 vol % CO_2_ was used to avoid respiratory alkalosis, and to be able to apply flat respiration with reduced movement. The vital functions and depth of anaesthesia were controlled throughout the experiment; the body temperature was monitored with the aid of a rectal temperature probe; ECG was recorded from the forelegs; O_2_ saturation of the blood and heart rate were monitored with pulse-oximeter (MouseOx STARR®, STARR Life Science Corp., Oakmont, PA). While the animal was paralyzed, several indications were taken as signs for sufficient depth of anaesthesia; the heart rate was kept at ~ 310 bpm, and did not accelerate during surgery; intrinsic temperature control failed, i.e. the animal had to be heated through a heating plate. For mechanical solidity, a flat tiltable pedestal was fixed with dental cement to the skull rostral to the bregma after removing the scalp. A circular trough (3 mm inner diameter) was milled into the skull, the centre of which was positioned so as to have the prospective window giving access to the visual cortex. The bony plate was then taken out along with the attached dura matter, and the arachnoid membrane was removed with a fine biology tipped forceps (Dumont #5 biology, Fine Science Tools GmbH, Heidelberg, Germany). A small tube was positioned at the edge of the hole in the skull to be able to extract excess cerebrospinal fluid. Care was taken not to damage the cortical surface and to avoid blood cell deposits at the region of interest. The window was sealed by a 5 mm diameter coverslip glued to the skull with tissue adhesive (Histoacryl®, BRAUN, Melsungen, Germany). If necessary, the excess cerebrospinal fluid was extracted so that the cortex had direct contact to the centre of the window. The lower surface of the coverslip was coated with a sparse layer of 40 nm fluorescent beads (yellow-green FluoSpheres™, Thermo Fisher Scientific, Waltham, MA) to render it visible in fluorescence contrast, and to control optical alignment. Coplanar alignment with respect to the focal plane was achieved by a laser beam reflected in two directions (90°) by the surface of the coverslip.

### *In vivo* STED microscope

We built a scanning STED microscope attached to an upright microscopy stand (Leica Microsystems GmbH, Wetzlar, Germany) similar to Willig *et al*.^20^. For excitation we used a pulsed laser diode operating at 490 nm and with 100 ps pulse duration (PiLas, Advanced Laser Diode Systems, Berlin, Germany). The STED beam was delivered by a Ti:Sapphire laser (MaiTai; Spectra-Physics, Santa Clara, CA) followed by an OPO (APE, Berlin, Germany) emitting 80 MHz pulses at 590 nm. The pulses were stretched to ~300 ps by dispersion in a glass rod and a 120 m long polarization-preserving fibre (OZ Optics, Ottawa, Canada). After passing through a vortex phase plate (RPC Photonics, Rochester, NY), the STED beam was co-aligned with the excitation beam by a custom made dichroic mirror and focused into the 1.3 NA objective lens (PL APO, 63x, glycerol; Leica, Wetzlar, Germany). The STED wavelength of 590 nm was chosen due to its closeness to the emission maximum of eGFP to provide an efficient off-switching. Shorter STED wavelengths would result in an excitation of eGFP and therefore in an unclear image. Images were recorded by beam scanning with a Yanus scan head (Till Photonics-FEI, Gräfelfing, Germany). The emitted fluorescence light was filtered with a 525/50 band-pass and focused on a multimode fibre for confocal detection connected to an avalanche photodiode (APD, PerkinElmer, Waltham, MA). To optimize the imaging parameters we varied the pixel dwell-time and laser powers. The imaging speed was increased to reduce the amount of STED photons per image. To compensate the low signal at the fast scanning speed we increased the excitation power. At 4–8 µs pixel dwell-time an optimum was reached where the detector started to saturate although the signal was still sufficient for a reliable STED image.

### Image collection

Z-stacks of 30 or 40 µm x/y STED microscopy images were recorded with Δz of 400 to 500 nm at different positions of the cranial window. The mouse was manoeuvred into position using an ASI motorised stage (MS-2000, Applied scientific instruments, Eugene, OR), which provided coordinates for each measurement. At each coordinate, a confocal x/z image was recorded using fluorescent beads adhered to the underside of the coverslip. This allowed for the accurate determination of the depth of the image plane. A confocal overview image was also taken as a reference point. Each coordinate was then assigned a time interval (1 min, 2 min, 3 min, 0.5 h, 1 h, 2 h, 3 h, 4 h, 5 h or 6 h, after which the stage was then manoeuvred to the respective coordinates. Using the overview image as a reference point, the focal area was then centred over the recurrent position and a STED microscopy image stack recorded. The images taken using the shorter 1 min time intervals were also used to examine the potential influence of the light on PSD95 assembly changes. All images were repeated 3–4 times before photobleaching limited the signal to noise ratio, making it impossible to re-image the same are.

Imaging parameters:

Figure 1: (A) P_Exc_ = 5.6 µW, DW = 4 µs, ΔX = ΔY = 30 nm

                  (B) P_Exc_ = 6.0 µW, P_STED_ = 35 mW, DW = 8 µs, ΔX = ΔY = 30 nm

Figures 2 and 3: P_Exc_ = 5.6 µW, P_STED_ = 21 mW, DW = 4 µs, ΔX = ΔY = 30 nm

P_Exc_: Average excitation power measured in the aperture of the objective. P_STED_: Average STED power measured in the aperture of the objective. DW: Pixel dwell time. ΔX, ΔY: Pixel size in x and y, respectively.

### Image processing

#### Particle analysis

The image analysis was performed using z-stack raw data taken from the Imspector software (Abberior Instruments, Göttingen, Germany) to create single plane and maximum intensity projection images. This data was then imported and investigated using Fiji^41^ with commands shown here in capital letters. Analyses were not made from the top and bottom planes of each z-stack, but these were utilised to ensure that all assemblies were at their maximal intensity within the remaining planes. These assemblies were first SMOOTHed and then analysed and evaluated for their size and shape. Here we used the OVAL SELECTION tool, using the SHIFT keyboard key, to encircle the smaller round spots with a continuous circle. Alternatively, elongated spots were marked by using either the STRAIGHT LINE or FREEHAND LINE tool, dependent on the straightness of the assembly, with large assemblies being marked using the FREEHAND SELECTION tool to ensure the whole assembly was included. The marked areas were then analysed (ANALYSE PARTICLES) using TOOLS and the Region Of Interest manager (ROI MANAGER). The freehand selections were further fitted with an ellipse by the Fiji implemented particle analyse tool, from which the major and minor axis was averaged to get an average diameter. The data was then exported from the ROI manager to a measurement sheet (MEASUREMENT) and further exported to Excel for subsequent comparative analysis.

#### Ensemble mapping

Images were analysed in Fiji. The z-stacks were SMOOTHed and a Z PROJECTION (maximum intensity) was generated for each time point. The MIPs of the 3 time points were concentrated to a stack (IMAGE TO STACK). BLEACH CORRECTION was executed and any small drift between the time points was corrected by aligning the images in x and y with STACKREG (AFFINE transformation). MAKE BINARY was used to generate binary images of the whole stack (Method OTSU). The binary stacks were transferred to Imspector software to display the 3 time points in RGB colour. For quantification of the extent of overlap the two binary images of the respective time points were summed up, yielding a value of two at overlapping positions, and a value of one at positions of the assembly which appear only at one or the other time point. The number of pixels of value two (N_2_) and the number of pixels of value one (N_1_) were then normalized:

$${\rm{Percent}}\,{\rm{of}}\,{\rm{pixel}}\,{\rm{not}}\,{\rm{changing}}:{\rm{nc}}={{\rm{N}}}_{2}/({{\rm{N}}}_{1}+{{\rm{N}}}_{2})\ast 100 \% $$

$${\rm{Percent}}\,{\rm{of}}\,{\rm{pixel}}\,{\rm{changing}}:{\rm{c}}={{\rm{N}}}_{1}/({{\rm{N}}}_{1}+{{\rm{N}}}_{2})\ast 100 \% $$

## Electronic supplementary material

Supplementary material
