# Supplementary material for: In vivo STED microscopy visualizes PSD95 sub-structures and morphological changes over several hours in the mouse visual cortex
Source: Sci Rep. 2018 Jan 9;8:219. doi: 10.1038/s41598-017-18640-z (PMC5760696; doi:10.1038/s41598-017-18640-z)
Supplement: Supplementary file 1 — Supplementary material [file 41598_2017_18640_MOESM1_ESM.pdf]

Supplementary Material

In vivo STED microscopy visualizes PSD95 sub-structures and morphological changes over several hours in the mouse visual cortex

Waja Wegner[1,2,3], Alexander C. Mott[1,3], Seth G.N. Grant[4], Heinz Steffens[1,2,3], Katrin I. Willig[1,2,3,*]

[1]Optical Nanoscopy in Neuroscience, Center for Nanoscale Microscopy and Molecular Physiology of the Brain, University Medical Center Göttingen, Göttingen, Germany
[2]Collaborative Research Center 889, University of Göttingen, Göttingen, Germany
[3]Max Planck Institute of Experimental Medicine, Göttingen, Germany
[4]Genes to Cognition Program, Centre for Clinical Brain Sciences, Chancellor's Building, University of Edinburgh, Edinburgh, EH16 4SB, UK

*Correspondence and requests for materials should be addressed to KIW: kwillig@em.mpg.de

Supplementary Figure

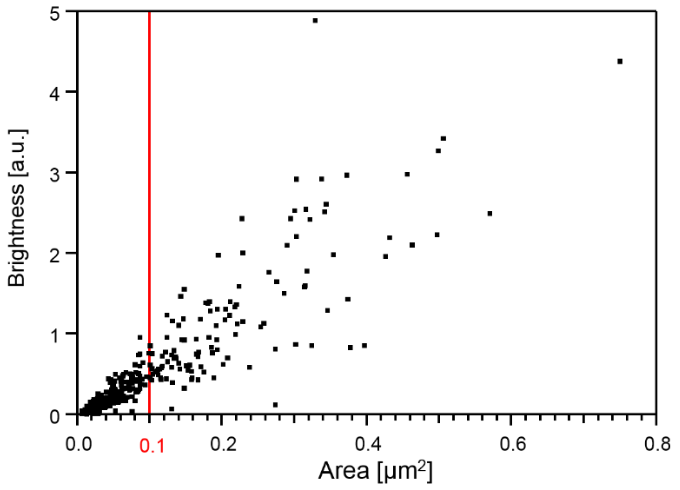

Figure S1: Brightness does not correlate with the size of the PSD95 assemblies. From all assemblies analysed for Figure 1 those chosen were most likely to be within the focal plane, i.e. all small spots or larger ones which were encircled for the analysis but not the elliptic assemblies which were measured with a line. The plot of the total fluorescence brightness over the area of the assembly shows that the brightness is not correlated with the area for areas above ~0.1 µm^2. Estimating the size of PSD95 assemblies from their brightness is therefore inaccurate.

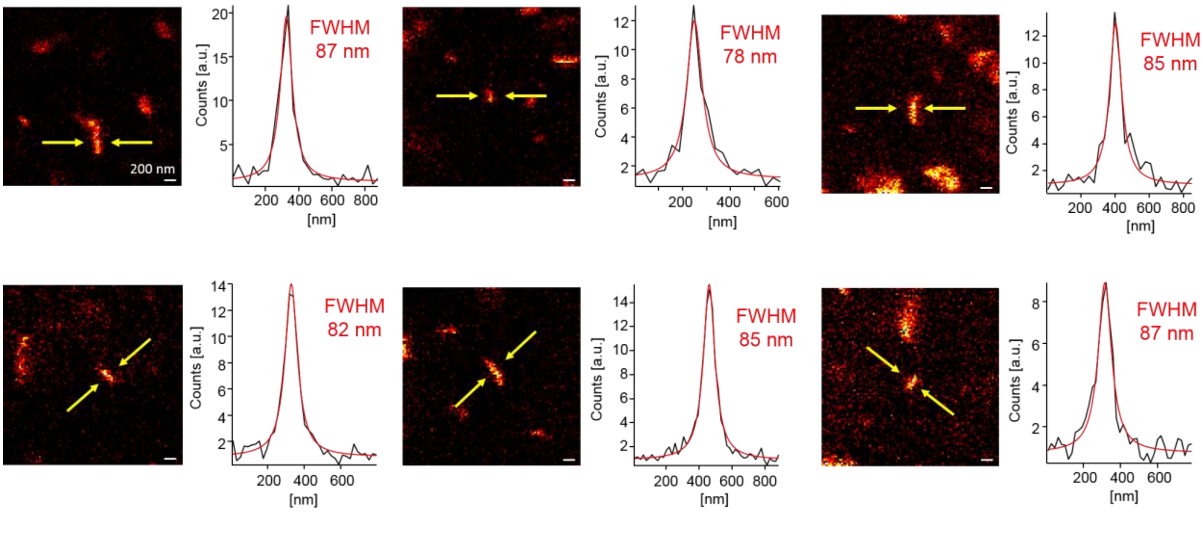

Figure S2: Resolving capability of the *in vivo* STED microscope on PSD95-eGFP. Crops of small PSD95 assemblies were chosen out of the whole STED microscopy image stack which includes Fig. 1b. Line profiles (black line) of an average of 3 lines at the indicated positions (arrows) of 6 different PSD95 assemblies were fitted with a Lorentzian function (red line). The full-width at half-maximum (FWHM) of the 6 fits average to 84 nm. 84 nm is an upper estimate of the resolving power of the microscope as it is a convolution of the PSD95 assembly of unknown size with the microscope's point spread function. However, this estimation includes all distortion which could occur by imaging in the tissue and in a living species and is therefore preferred to a calibration with fluorescent beads on the cover glass or a calibration with eGFP expression in cultured cells.

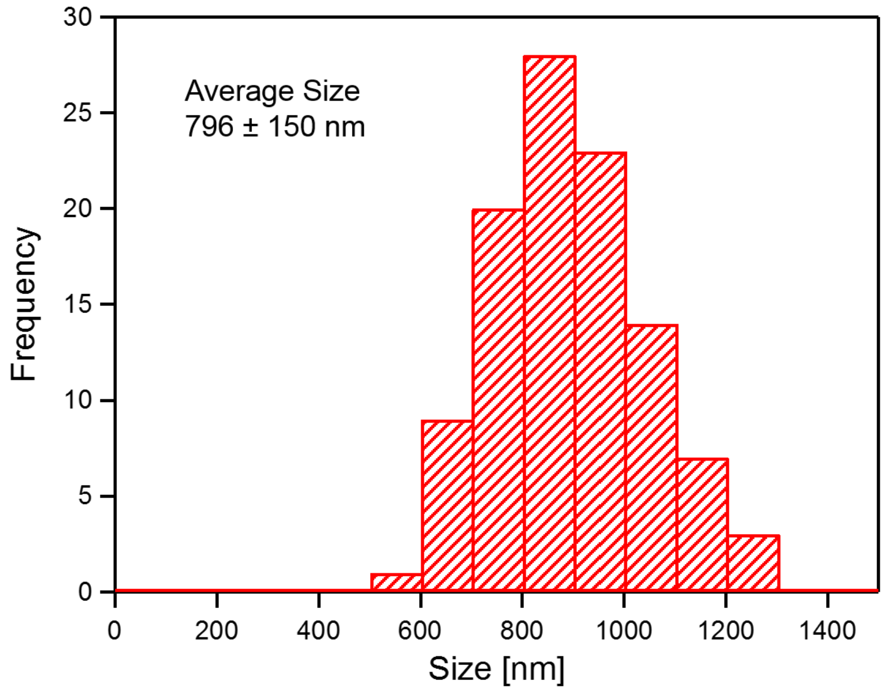

Figure S3: Absolute frequency histogram of the size of all large assemblies analysed in Figure 2E. Analysis was performed equivalent to Figure 1E. Average size ± SD

Supplementary Data

Δt=1 min

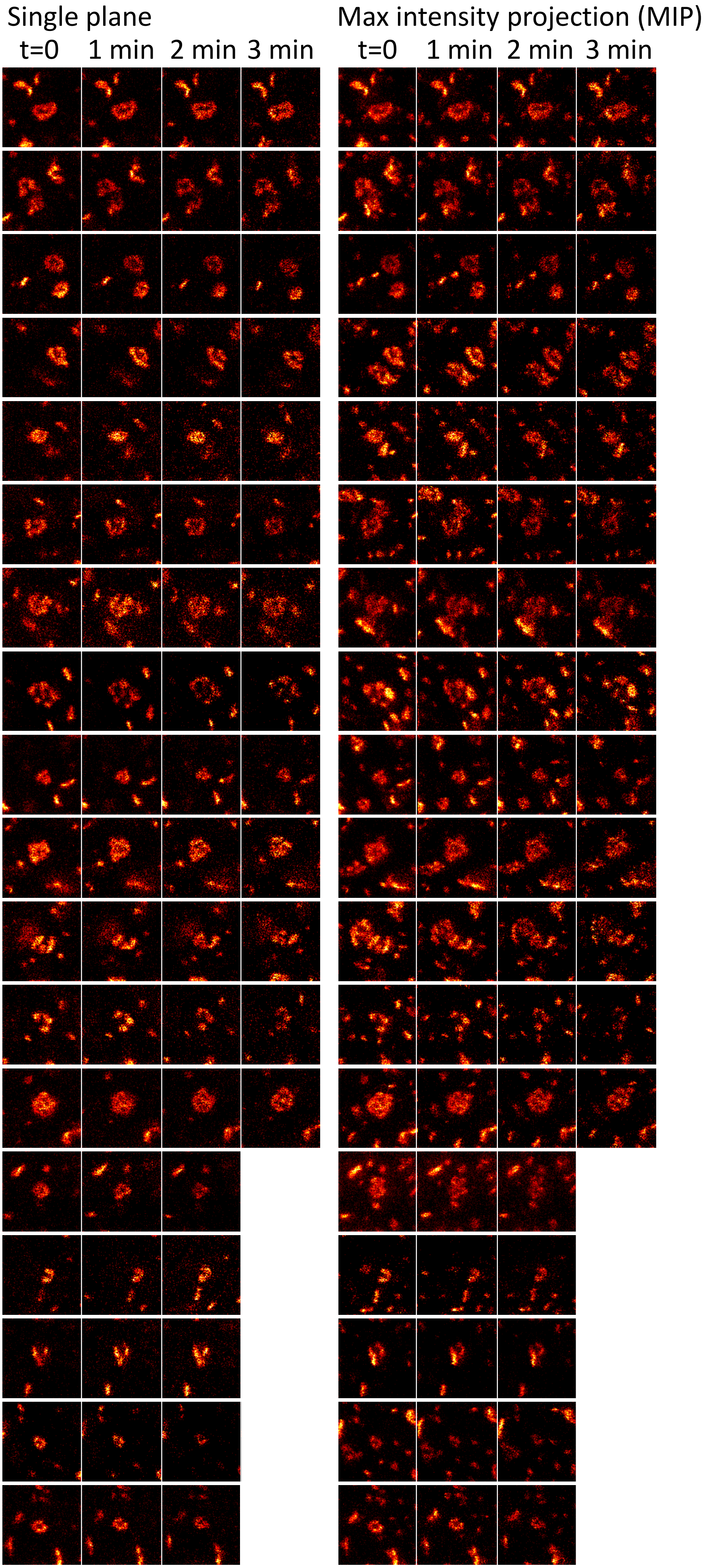

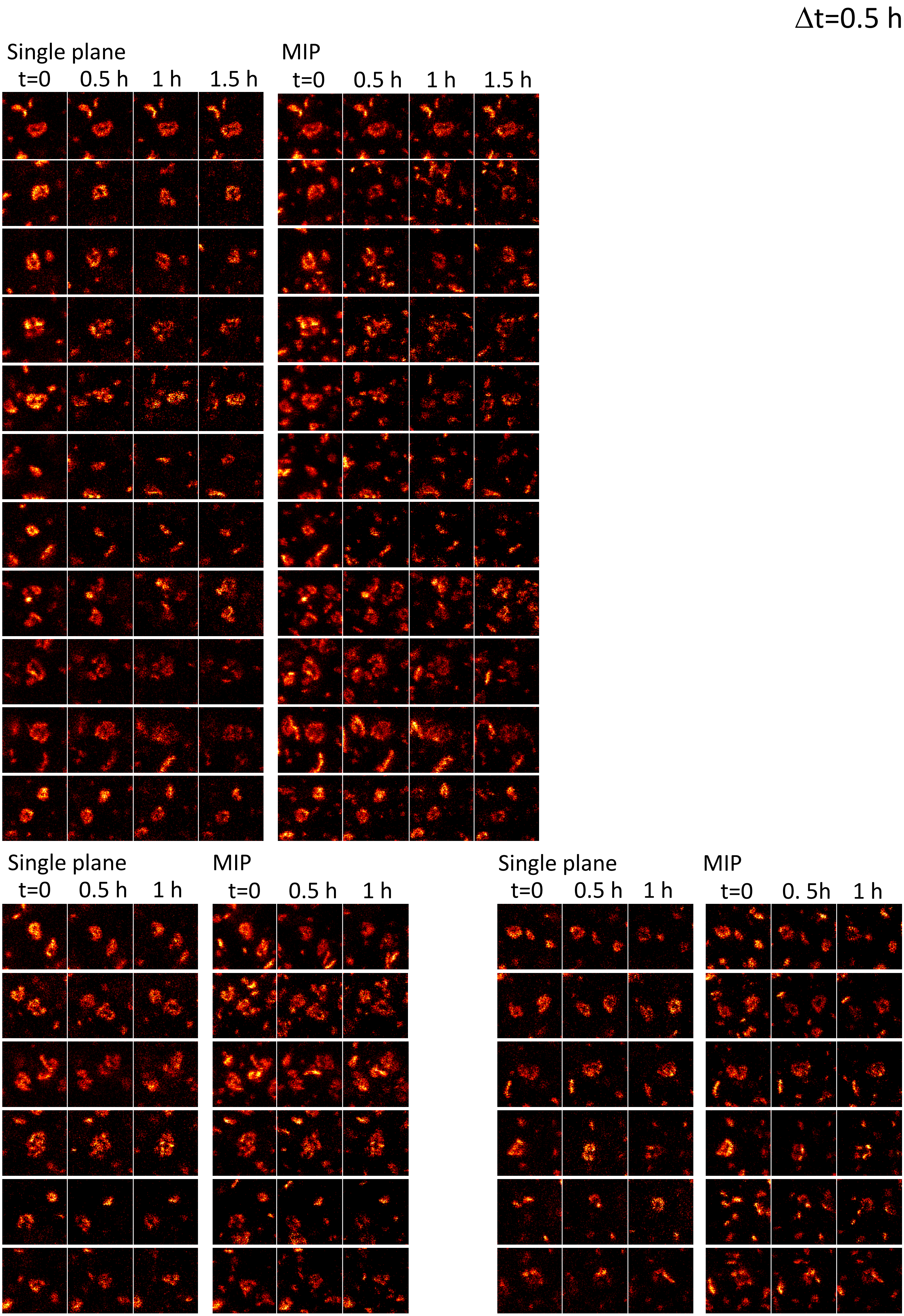
Δt=0.5 h
Single plane
t=0
0.5 h
1 h
1.5 h
MIP
t=0
0.5 h
1 h
1.5 h
Single plane
t=0
0.5 h
1 h
MIP
t=0
0.5 h
1 h
Single plane
t=0
0.5 h
1 h
MIP
t=0
0. 5h
1 h

Δt=0.5 h

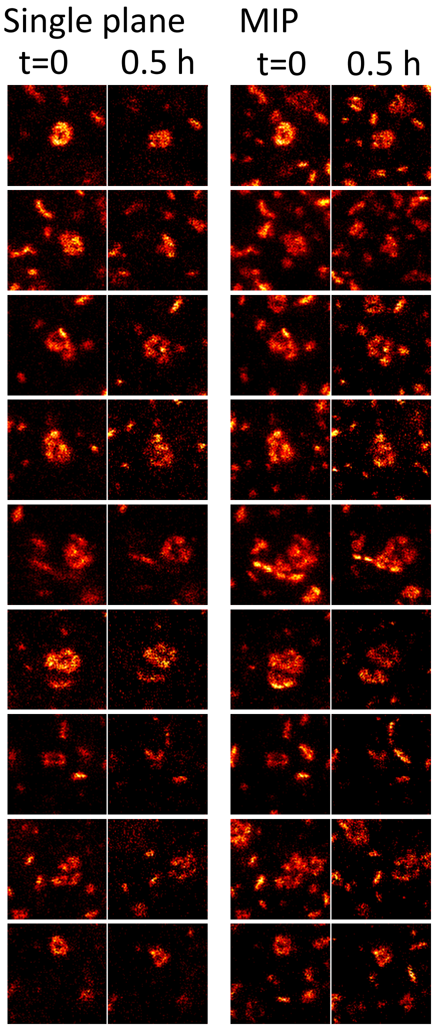
Single plane
t=0
0.5 h
MIP
t=0
0.5 h

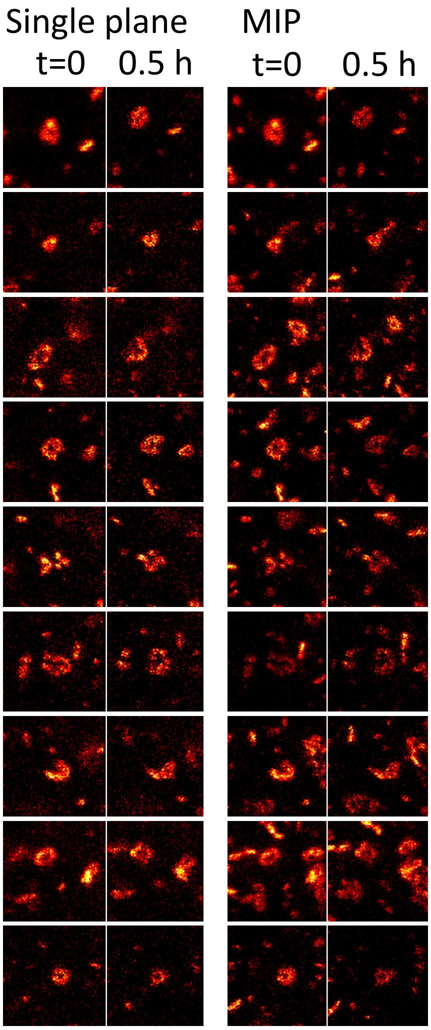
Single plane
t=0
0.5 h
MIP
t=0
0.5 h

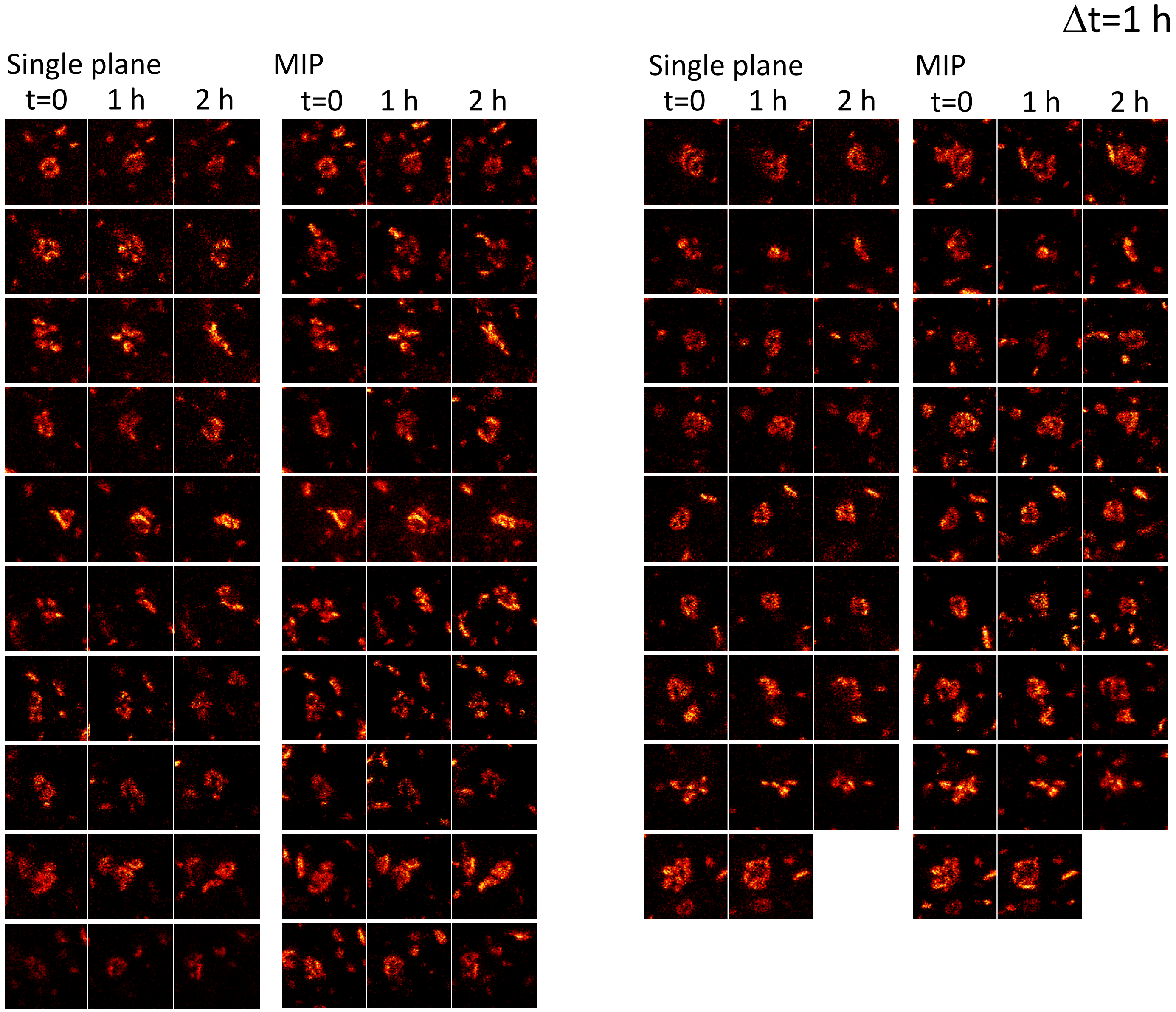
Δt=1 h
Single plane
t=0
1 h
2 h
MIP
t=0
1 h
2 h
Single plane
t=0
1 h
2 h
MIP
t=0
1 h
2 h

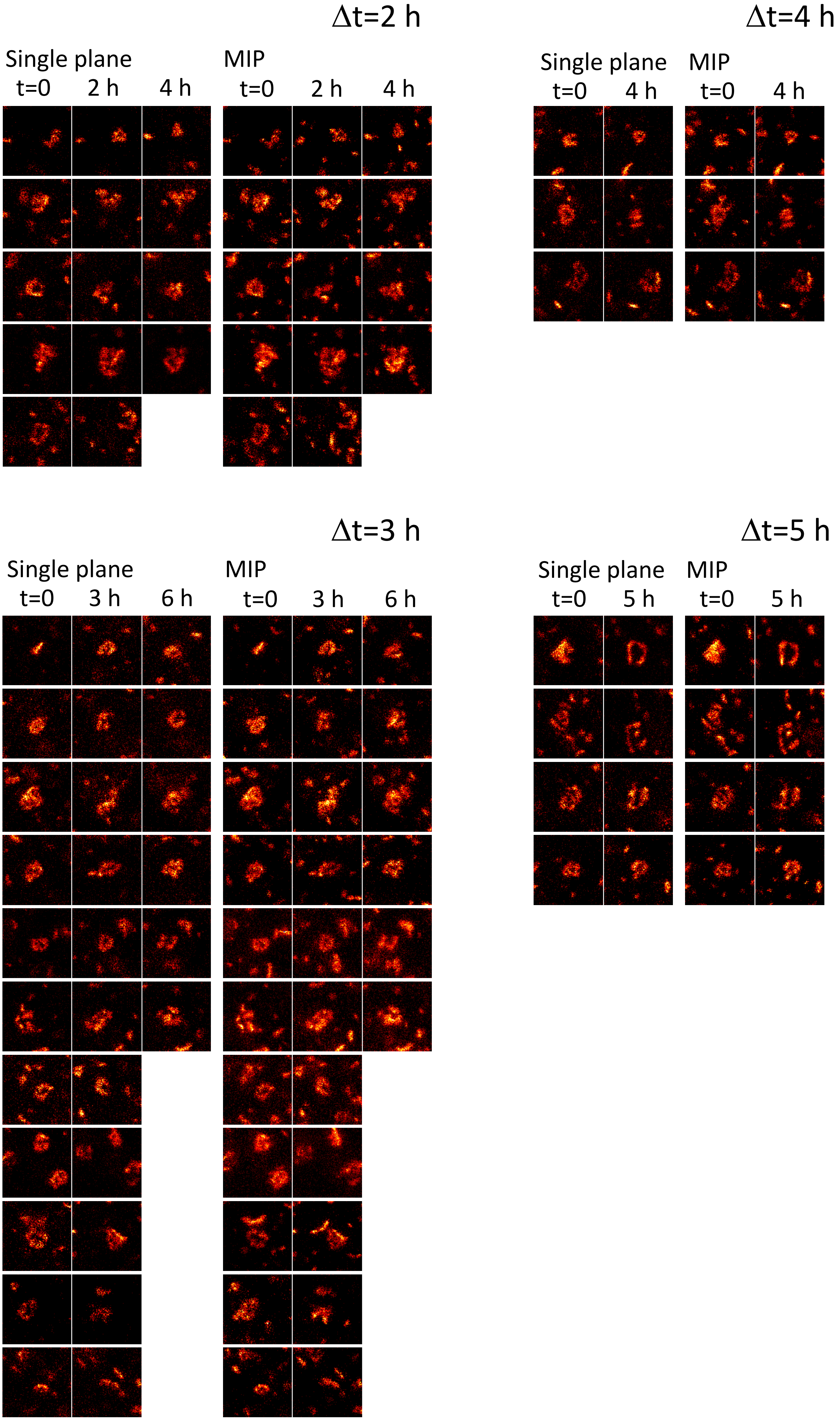

Δt=2 h
Single plane
t=0
2 h
4 h
MIP
t=0
2 h
4 h
Δt=4 h
Single plane
t=0
4 h
MIP
t=0
4 h
Δt=3 h
Single plane
t=0
3 h
6 h
MIP
t=0
3 h
6 h
Δt=5 h
Single plane
t=0
5 h
MIP
t=0
5 h
